# Title: role of matrix metalloproteinase −9 in progression of tuberculous meningitis: a pilot study in patients at different stages of the disease

**DOI:** 10.1186/s12879-016-1953-9

**Published:** 2016-11-29

**Authors:** S. Majeed, P. Singh, N. Sharma, S. Sharma

**Affiliations:** 1 Department of Biochemistry, Postgraduate Institute of Medical Education & Research, Chandigarh, 160012 India; 2 Department of Neurology, Postgraduate Institute of Medical Education & Research, Chandigarh, 160012 India; 3 Department of Internal Medicine, Postgraduate Institute of Medical Education & Research, Chandigarh, 160012 India

**Keywords:** Antitubercular drugs, Inflammation, *Mycobacterium tuberculosis*, Matrixmetalloproteinase-9, SB-3CT, Tissue inhibitor of metalloproteinase-1, Tuberculous meningitis

## Abstract

**Background:**

TBM (Tuberculous meningitis) is severe form of tuberculosis causing death of one third of the affected individuals or leaving two-third of the survivors disabled. MMP-9 (Matrix metalloproteinase-9) is produced by the central nervous system in a variety of inflammatory conditions and has a role in the breakdown of extracellular matrix and blood–brain barrier.

**Methods:**

In this study, the levels of MMP-9 and its inhibitor, TIMP-1 (tissue inhibitor of metalloproteinases-1), were screened using zymography and reverse zymography in cerebrospinal fluid and serum of tuberculous meningitis patients at different stages of the disease. Further, role of MMP-9 as therapeutic target was studied in C6 glioma cells infected with *Mycobacterium tuberculosis* H_37_R_v_. Cells were treated with dexamethasone or SB-3CT (specific inhibitor of MMP-9) in combination with conventional antitubercular drugs.

**Results:**

MMP-9 levels in patients were increased as the disease progressed to advanced stages. The infection led to increased MMP-9 levels in C6 glioma cells and specific inhibition of MMP-9 by SB-3CT augmented bacillary clearance when used along with antitubercular drugs.

**Conclusion:**

MMP-9 plays a prominent role in progression of tuberculous meningitis from initial to advanced stages. Increased levels of MMP-9 during advancement of the disease leads to degeneration of nervous tissue and blood brain barrier disruption. Hence, MMP-9 can be considered as a therapeutic target for efficient management of TBM and can be explored to inhibit further progression of the disease if used at an early stage.

## Background

Tuberculous meningitis is the highest mortality causing form of tuberculosis, one third of the patients die and more than half of survivors are left disabled after contracting the disease [1, 2]. Severe clinical manifestations of tuberculous meningitis occur due to robust inflammatory response generated in the brain against pathogenic bacilli [3]. During this process, microglial cells are activated to secrete proteases which have ability to degrade extracellular matrix and cause tissue destruction [4, 5]. Among these proteases, MMPs seem to have prominent role in tissue destruction. MMPs are endopeptidases which degrade all the components of the extracellular matrix and thus have potential to disrupt blood brain barrier and cause CNS damage [5]. Tuberculosis infection elicits the production of MMP-9 by direct interaction between cell wall components of bacilli, human monocytes and macrophages.[6] The higher increase of MMP-9 in pleural effusions of tuberculous meningitis patients than in patients with malignant pleural disease also signifies the role of MMP-9 in tuberculous infections and vice versa [7]. Besides, the synergistic effects of MMP-9 and Mycobacteria on each other, specific MMP-9 substrates type IV collagen and laminin, constitute essential structural components of the blood brain barrier reflecting its importance in tissue damage. The possibility of using MMP-9 inhibition as therapeutic target can be considered for preventing progression of disease to advanced stages and control disabilities. Currently corticosteroids like dexamethasone or prednisolone are usually given along with conventional antitubercular drugs to combat the inflammatory tissue degradation which nonspecifically inhibits MMP 9 as well [8, 9]. Specific inhibitor of MMP-9, SB-3CT has come forth with desirable properties of crossing blood brain barrier and therapeutic effects for treating neurological diseases due to inflammation [10]. This inhibitor has been studied for ischemia of immature brain and found to work efficiently; showing its utility in pediatric cases [11]. In this study, role of MMP-9 has been evaluated in progression of TBM. The levels of MMP-9 at different stage of the disease were compared to evaluate if any correlation exists between the worsening prognosis and MMP-9 levels. Though the inflammatory tissue destruction amplifies as the disease progresses from stage I to stage III, no study is available on the exact contribution of these MMPs in advancement of the disease. Effect of specific inhibition of MMP-9 on clearing the bacterial burden were further evaluated in C6 glioma cells infected with *Mycobacterium tuberculosis* H_37_R_v_.

## Methods: availability of data and material section

### Ethical considerations

The plan of work was approved by Institutional Ethics Committee (IEC) of Postgraduate Institute of Medical education and Research, Chandigarh, and sample collection was done according to the ethical guidelines. CSF and blood samples were used after written consent from the patients or their attendants in case of unconscious or confused patients. The written consent was recorded in patient proforma approved by Institutional Ethical Committee.

### Chemicals and consumables

Isoniazid, Rifampicin, Pyrazinamide, Dexamethasone, MMP-9 standard, MMP-9 inhibitor (SB-3CT) and gelatin were obtained from Sigma (St. Louis USA). OADC (Oleic albumin dextrose catalase) enrichment and 7H11 agar were obtained from Difco-Becton-Dickinson (USA), FBS (fetal bovine serum) and MEM (minimum essential medium) were obtained from Invitrogen Corporation (Gibco), New York, USA. Acrylamide, bisacrylamide, ammonium persulphate and other reagents used were of molecular grade. Ultrapure water was used throughout the study.

### Mycobacterial culture and cell lines

*Mycobacterium tuberculosis* H_37_R_v_ (NCTC7416) was originally obtained from National Collection of Type Culture (NCTC) London, UK. Bacteria were grown in sterile Sautons’s medium and maintained in sterile lowenstein-jensen medium. Cultures were grown under shaking conditions at 180 rpm, 37 °C.

### Cell lines

C6 glioma and HT1080 cell lines were purchased from National Centre for Cell Science, Pune. C6 glioma cells were grown in MEM supplemented with 10% FBS, HT1080 cells were also grown in MEM supplemented with 10 mM sodium pyruvate and 10% FBS, grown at 37 °C in a humidified incubator under 5% C0_2_ and 95% air. Cells were maintained as frozen aliquots of 10% DMSO in FBS at −80 °C. Conditioned media containing gelatinases was obtained by growing HT-1080 cells initially in MEM supplemented with 10% FBS and then in serum-free MEM for 24 h.

### Study design, site and population

Study subjects included patients with TBM attending Neurology, Emergency or Internal medicine departments at PGIMER, Chandigarh, India. Patients above 12 years of age and of either sex were included in the study. Patients infected with HIV or suffering from any other infectious disease in addition to TBM were excluded from the study. The plan of work was approved by Institutional Ethics Committee of Postgraduate Institute of Medical Education and Research, Chandigarh and sampling was carried out according to the ethical guidelines. CSF and blood samples were used after written consent from the patients or their attendants in case of unconscious or confused patients. Samples were taken during treatment of patients. The written consent was recorded in patient proforma, which was approved by Institutional Ethical Committee. Performa was used to collect the demographic and clinical information, including age, sex, HIV status, duration, regimen, tests and examinations of previous treatment, etc. Ninety-one patients with clinical symptoms of TBM and 16 control subjects were included in the study. The selection of patients was based on the criteria defined by Mahadevan et al. [12] i.e. fever over a period of week, high protein sugar ratio in cerebrospinal fluid, imaging studies of head showing exudates, hydrocephalus, infarcts, cranial nerve palsies, response to antitubercular drug therapy and final confirmation of tuberculous meningitis by the clinician attending the patient. According to the guidelines of British Medical Research Council (MRC), the patients were categorized into three stages I, II and III as per the symptoms shown by the patients. Stage I included conscious and rational patients with no signs of focal or neurological deficits or neck stiffness, Stage II patients were conscious but confused had focal signs such as cranial nerve palsies or hemiparesis. Patients in stage III were comatose or delirious with or without dense neurological deficit. Controls subjects were those having non inflammatory disease. CSF and blood samples were obtained from patients in different stages of TBM (10 patients with stage I, 42 with stage II, 39 with stage III) and 16 control subjects. CSF sample was obtained by lumbar puncture during their hospitalization and blood sample (5 ml) was drawn in sterile tube and processed for serum separation. CSF and serum samples were sterile filtered and immediately frozen at −80 °C till assayed for MMP-9 and TIMP-1 levels.

### Gelatin-substrate zymography

MMP-9 levels in CSF and serum were monitored by gelatin zymography using 8% polyacrylamide gels containing 0.3% SDS and gelatin (1 mg/ml) [13]. Undiluted CSF and serum (1:100 dilutions) was mixed with zymography buffer [50 mM Tris–HCl, 10% glycerol, 2% SDS and 0.01%bromophenol blue]. Samples were loaded and run at 20 mA for 20 min and then at 30 mA for 60 min. Equal amount of each sample was used for analysis. Authentic MMP-9 standard was also run as control. Gels were agitated in 2.5% TritonX-100 and washed in 50 mM Tris–HCl buffer, pH 7.5 containing 200 mM NaCl. Thereafter, gels were incubated overnight at 37 °C in renaturing buffer (50 mM Tris–HCl buffer, pH 7.5 containing 200 mM NaCl, 5 mM CaCl_2_, 0.02% (w/v) brij-35, and 0.01% sodium azide. Finally, gels were stained with Comassie G blue and or silver stained to obtain the bands. Intensities of gelatinolytic bands corresponding to MMP-9 were measured by software analysis [13, 14] The densitometric intensities of known concentrations of MMP-9 standard were plotted to obtain standard curve from which MMP-9 concentrations in patient samples was determined.

### Reverse zymography

TIMP-1 in CSF and serum samples was analyzed by reverse zymography. 15% polyacrylamide gels with gelatin and gelatinases copolymerized into the matrix were prepared. Gelatinases were obtained from serum free media of HT1080 fibrosarcoma cell line. Samples were mixed with zymography buffer and run for electrophoresis. Gels were agitated in 2.5% TritonX-100, washed in 50 mM Tris–HCl buffer, and incubated overnight in renaturation buffer for action of gelatin and gelatinases. During the activation step, gelatinases present in gel digest the gelatin but only in areas where TIMPs are absent. Upon staining, the levels were monitored as blue bands of undigested gelatin on a clear gelatinolytic background [15].

### Effect of MMP-9 inhibition on C6 glioma cells infected with *Mycobacterium tuberculosis*

C6 glioma cells were plated in 24 well plates and infected with *Mycobacterium tuberculosis* H_37_R_V_ in ratio of 10 bacilli per cell. After 90 min., the cells were washed extensively to remove extracellular bacteria. Cells were divided into seven groups Group I: uninfected cells, Group II: cells infected with *Mycobacterium tuberculosis* H_37_R_V_, Group III: infected cells treated with dexamethasone, Group IV: infected cells treated with MMP-9 inhibitor (SB- 3CT), Group V: infected cells treated with antitubercular drugs, Group VI: infected cells treated with antitubercular drugs along with dexamethasone, Group VII: infected cells treated with antitubercular drugs along with SB-3CT. Concentration of drugs, dexamethasone and SB-3CT used was as described [16–18]. After 5 days of treatment, cells were lysed with 0.1% SDS. Mixture of extra cellular media and cell lysate was processed for CFU enumeration and MMP-9 analysis.

### Statistical analysis

Comparison of MMP-9 levels amongst patients in different stages of TBM was performed using Kruskal-Wallis test. If the overall test was significant (*p* < 0.05) the Mann–Whitney test was applied to compare control subjects with stage I, II, III patients; stage I patients with stage II, III patients and stage II patients with stage III patients. All the comparisons were based on mean rank.

## Results

### Levels of MMP-9 and TIMP-1 in CSF of patients with advanced stages of TBM

**Fig. 1:**
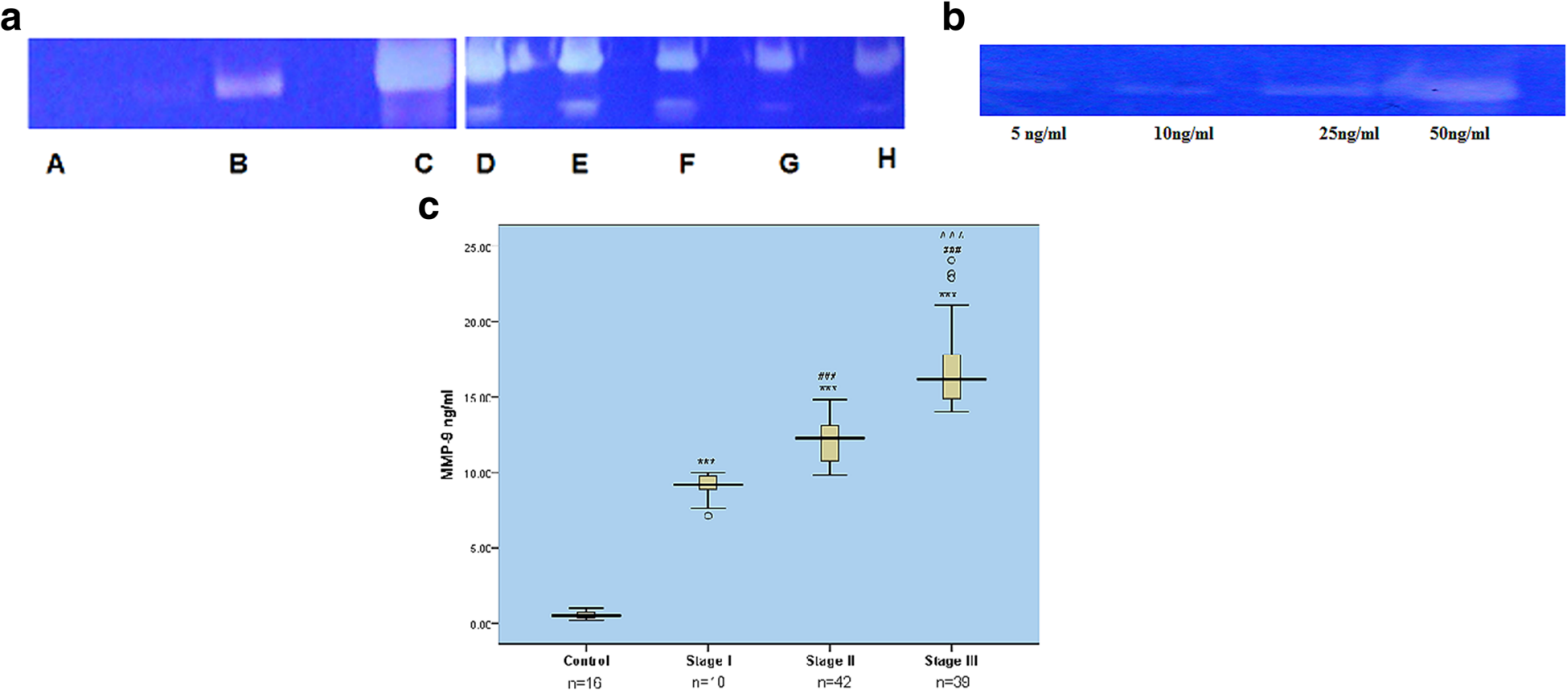
**a** Representative zymograms showing MMP-9 in CSF: *Lane A:* control, *Lane B:* Stage I, *Lane C:* authentic MMP-9, *Lanes D, E:* stage III; *Lanes F, G, H:* stage II. Equal amount of sample was loaded in each lane (**b**) Zymogram of known concentrations of standard MMP-9 (**c**) Box plot showing MMP-9 levels in CSF of patients in different stages of tuberculous meningitis***p < 0.001 compared to control, ### *p* < 0.001 compared to stage I and control, ^^^ *p* < 0.001 compared to stage II

**Fig. 2:**
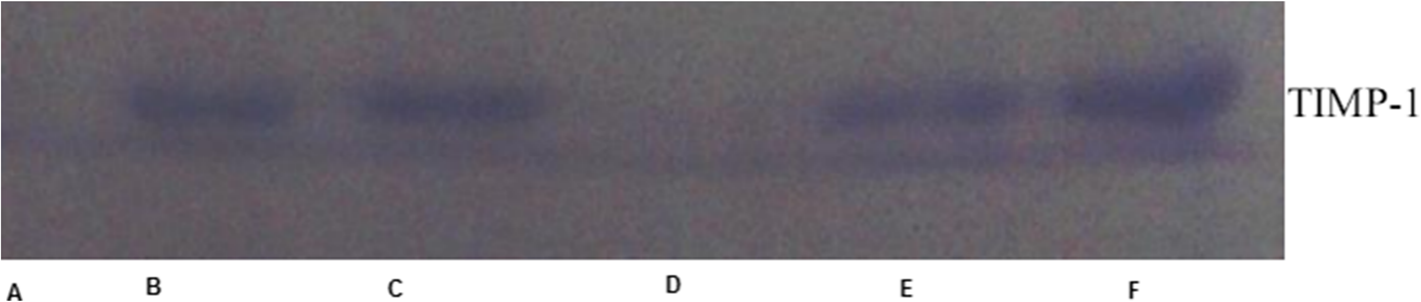
Representative reverse zymogram of TIMP-1 in CSF: *Lanes A:* stage II; *Lanes B, C:* stage I; *Lane D*, stageIII; *Lanes E, F:* control subjects. Equal amount of sample was loaded in each lane

TBM patients selected for the study were grouped into stage I, II and III as per details given in Table 1. The amount of MMP-9 in CSF samples was quantified by comparing their densitomertic values with those of known concentration of MMP-9 standard. CSF zymography revealed that MMP-9 levels increased with advanced stages of TBM (Fig. 1a). MMP-9 levels were 0.62 ± 0.40 ng/ml for controls, 9.0 ± 0.87 ng/ml for stage I, 12.0 ± 1.34 ng/ml for stage II and 16.86 ± 2.7 ng/ml for stage III tuberculous meningitis patients (Fig. 1b and c). MMP-9 levels showed significant increase in samples of stage III patients as compared to stage I and stage II. TIMP-1 was detectable in stage I TBM patients only. Out of 10 stage I patients, it was detected in only 3 patients. However, TIMP-1 was detected in all control samples but no sample from stage II or III patients showed the presence of TIMP-1 (Fig. 2).

**Table 1:** Demography data of the subjects included in the study Patients in advanced stages of the disease showed higher protein and lower glucose content in their CSF

	No. of individuals	Average age (years)	Male:Female	CSF Protein (mg/ml)	CSF glucose (mg/ml)
Stage 1	10	36.75±14.1	6:4	121.7±21.5	32.3±6.5
Stage 11	42	34.66±15.5	24:18	135±44.5	31.8±16.3
Stage III	39	53.6±10.5	23:16	245±89.2	25.72±7.9
Control	16	35.93±8.3	10:6	21.2±6.5	63.88±11.3

### Levels of MMP-9 and TIMP-1 in serum samples of patients with advanced stages of TBM

**Fig. 3:**
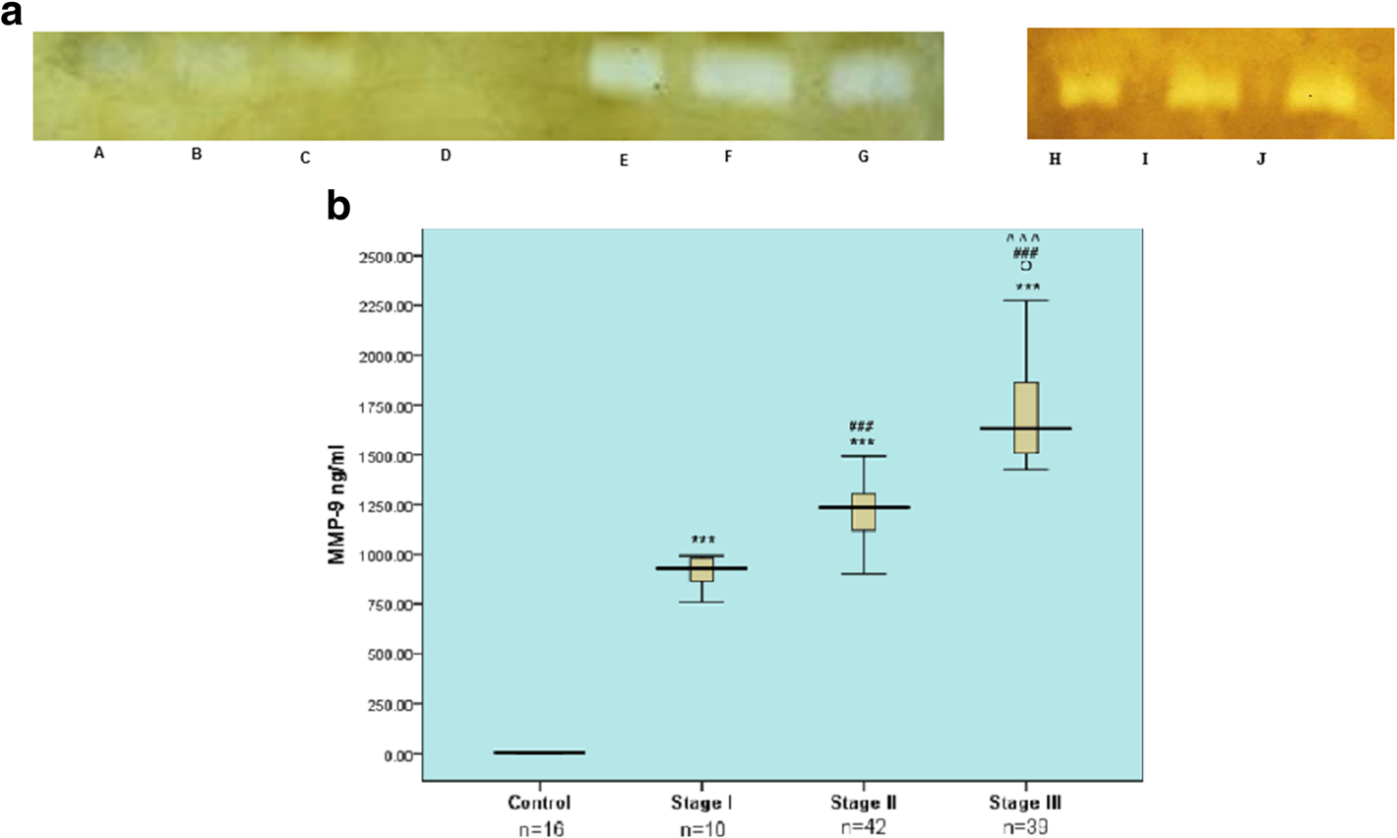
Representative zymogram of MMP-9 in serum samples of tuberculous meningitis patients. **a** *Lanes A, B, C:* stage I; *Lane D:* control subject; *Lanes H, I, J:* stage II. **b** Box plot showing MMP-9 levels in serum of patients in different stages of TBM ***p < 0.001 compared to control, ### *p* < 0.001 compared to stage I and control, ^^^ *p* < 0.001 compared to stage II

Increase in MMP-9 levels with advancement of the disease was observed in serum samples hence confirming the results obtained with CSF (Fig. 3a). Densitometric analysis revealed MMP-9 levels of 5.67 ± 2.45 ng/ml for controls, 830.66 ± 83.07 ng/ml for stage I, 1202.55 ± 136.81 ng/ml for stage II and 1679 ± 277.4 ng/ml for stage III TBM patients (Fig. 3b). TIMP-1 was not detected in serum sample of any subject.

### Evaluation of MMP-9 as therapeutic target using SB-3CT (MMP-9 inhibitor) and dexamethasone in C6 glioma cells infected with *Mycobacterium tuberculosis* H_37_R_v_

**Fig. 4:**
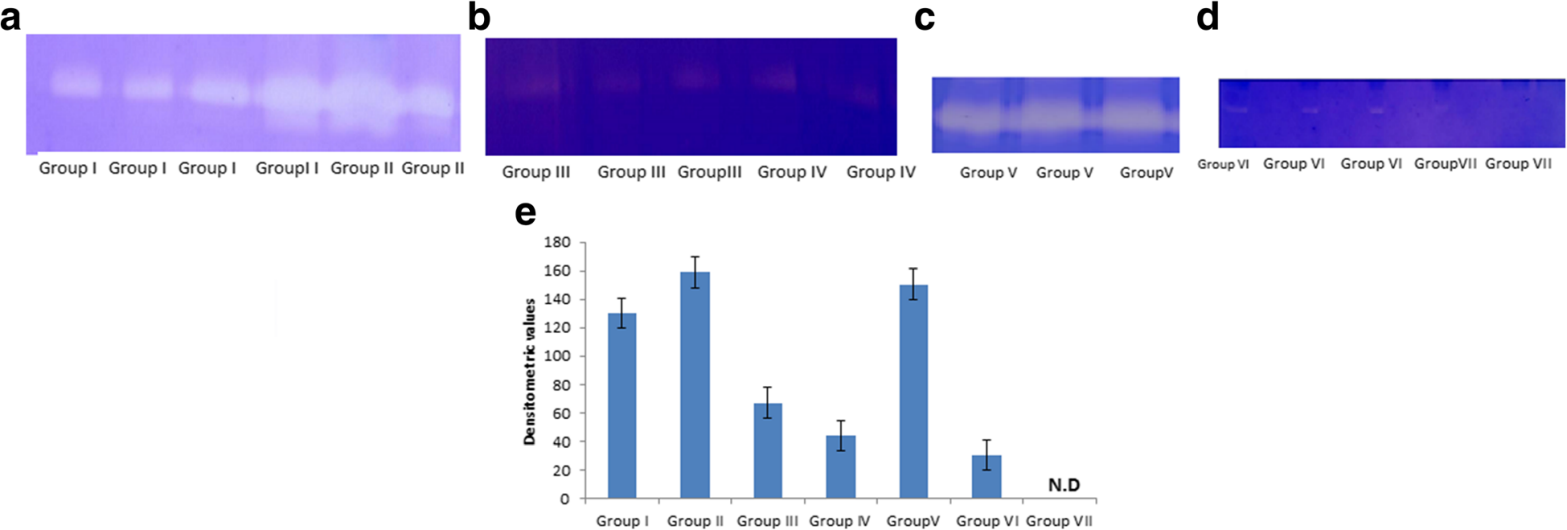
Zymograms of MMP-9 levels in C6 cells (**a**) *Group I*: uninfected and *Group II:* infected cells (**b**) *Group III:* dexamethasone treated and *Group IV:* SB-3CT treated cells (**c**) Group V:treated with antitubercular drugs only (**d**) *Group VI:* treatment with antitubercular drugs along with dexamethasone and *Group VII:* treatment with antitubercular drugs along with SB-3CT (**e**) Graphical representation of Densitometric units corresponding to MMP-9 bands in different treatment groups of C6 Glioma Cells

**Fig. 5:**
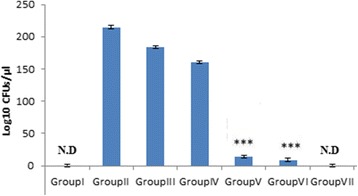
Log_10_ CFUs of *Mycobacterium tuberculosis* H_37_R_v_ infected C6 glioma cells after 5 days of treatment. *Group I:* uninfected cells, *Group II:* Untreated C6 cells infected with *Mycobacterium tuberculosis* H_37_R_v,_ *Group III:* Infected C6 cells treated with Dexamethasone, *Group IV:* Infected C6 cells treated with SB-3CT, *Group V:* Infected C6 cells treated with antitubercular drugs (Isoniazid + Rifampicin + Pyrazinamide) *Group VI:* Infected cells treated with antitubercular drugs along with dexamethasone, *Group VII:* Infected cells treated with antitubercular drugs along with SB-3CT. Values are mean ± SD of cells processed in triplicate. *** *p* < 0.001, respect to *Group II*

Levels of MMP-9 were increased in *Mycobacterium tuberculosis* infected C6 cells as compared to the uninfected cells (Fig. 4a). Ex vivo study can mimic the in vivo model of *Mycobacterium tuberculosis* infection where MMP-9 levels are known to be elevated due to infection. After confirming the elevation of MMP-9 levels in infected cells, the effect of MMP-9 inhibitor (SB-3CT) and dexamethasone was monitored in the infected C6 glioma cells. Levels of MMP-9 were reduced to greater extent after treatment with either SB-3CT or dexamethasone (Fig. 4b). Levels of MMP-9 remained unaffected when treated with antitubercular drugs alone (Fig. 4c.) and were markedly reduced when drugs were given along with dexamethasone or SB-3CT, (Fig. 4d). Cells treated with only SB-3CT or dexamethasone did not show decrease in CFUs. Interestingly, cells when treated with antitubercular drugs along with dexamethasone or SB-3CT, showed almost undetectable CFUs after 5 days of treatment (Fig. 5).

## Discussion

**Fig. 6:**
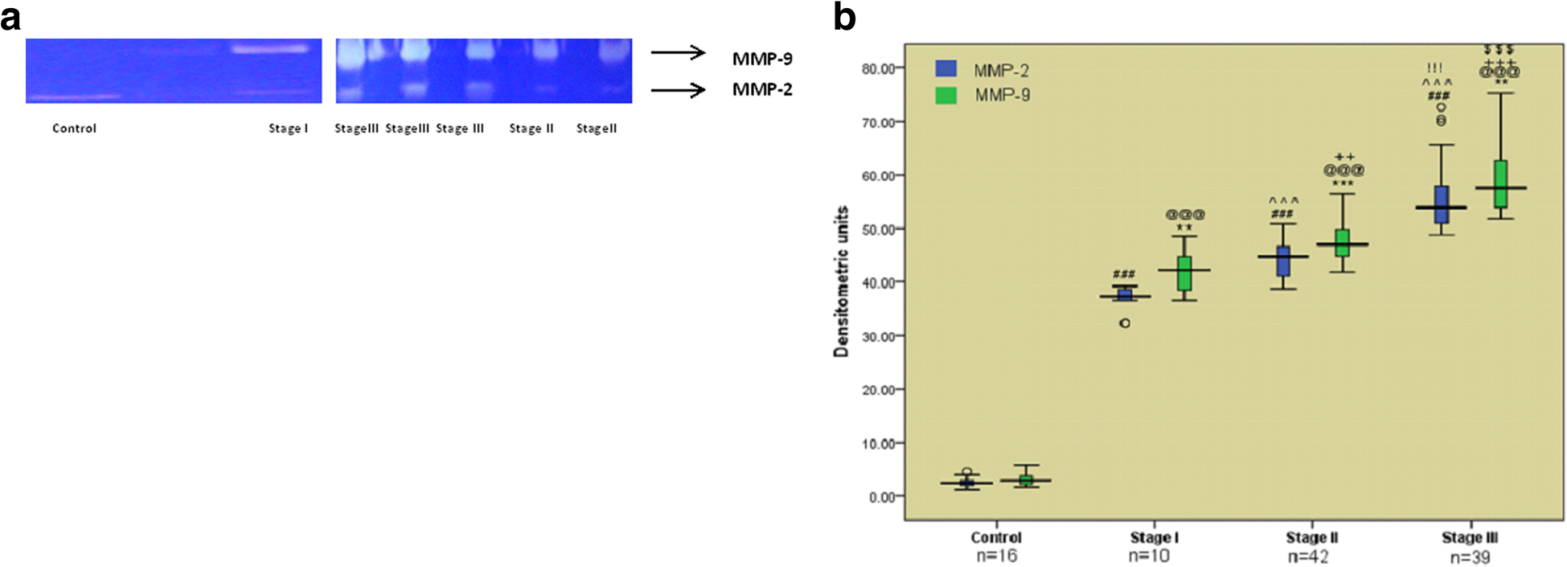
**a** Representative zymograms showing Low MW gelatinase in different stages of TBM. **b** Box plot showing comparison of densitometric values and low MW gelatinase in samples of control subjects and TBM patients

In this study, MMP-9 in CSF as well as serum was increased with progression of disease from stage I to III. Though increased levels of MMP-9 in TBM has been reported earlier [13, 19] this study compared the concomitant increase in MMP-9 levels with progression of the disease from earlier to its advanced stages. It was found that MMP-9 levels were significantly increased as disease progressed from stage I to II and significant increase was seen as the disease progressed to stage III. Other low MW gelatinase was also detected which is predicted to be MMP-2 based on its position on the gel. The concomitant increase of this gelatinase was also found with the advancement of the disease but increment in MMP-9 levels was significantly higher (Fig. 6a and b). Association of MMP-9 has been observed in necrotic meningeal vessels in TBM. CSF concentrations of MMP-9 has been correlated with involvement of brain ischaemia in TBM [20]. MMP-9 levels could not be detected in control samples while the low MW gelatinase was detected in these samples demonstrating that it is constitutively expressed. TIMP-1 levels in CSF samples were decreased with advancement of the disease and were undetectable in serum samples. Thus overall ratio of MMP-9/TIMP-1 was found to be increased leading to matrix degrading phenotype. Studies have found generation of matrix-degrading phenotype in tuberculosis and implicate MMP-9 as key mediator in tuberculosis pathology [21]. Other studies have reported uncontrolled rise of MMP-9 in CNS TB due to insignificant levels of TIMP-1 in comparison to MMP-9 which results in matrix degrading phenotype [22]. Role of *Mycobacterium tuberculosis* infections in elevating MMP-9 levels has also been documented [23]. Thus TBM seems to have dual relation with MMP-9. MMP-9 is involved in inflammatory response to disease and on the other hand *Mycobacterium tuberculosis* is involved in increasing MMP-9 levels. The data supporting the inflammatory as well as pathological role of MMP-9 in tuberculosis reveals the possibility of its prominent therapeutic role. This pilot study demonstrates the apparent role of MMP-9 in TBM pathology. Further, *Mycobacterium tuberculosis* infection enhanced levels of MMP-9 in C6 glioma cells as explained in earlier studies [24] wherein, conditioned media from *Mycobacterium tuberculosis* infected monocytes induced MMP-9 secretion from astrocytes. MMP-9 inhibitor SB-3CT and dexamethasone was used to inhibit *Mycobacterium tuberculosis* induced MMP-9 in C6 glioma cells. MMP-9 levels were decreased in cells treated with SB-3CT or dexamethasone. These results are in agreement with earlier studies [25]. Levels of MMP-9 were not apparently affected with antitubercular drugs only indicating that antitubercular drugs are not involved in reducing inflammation in tuberculous meningitis patients which explains the life-long neurological defects even after bacillary clearance. When drugs were used along with SB-3CT or dexamethasone, the MMP-9 levels were decreased to almost undetectable levels. CFU counts were significantly decreased upon treatment with antitubercular drugs. Bacillary counts were further decreased when SB-3CT or dexamethasone was used along with antitubercular drugs. No report about combined effects of antitubercular drugs with SB-3CT is available in the literature. MMP-9 seems to be a promising molecule to resolve the destructive outcome of TBM; however, further studies are required to assign therapeutic significance to MMP-9 inhibition for better management of TBM.

## Conclusions

The present work suggested important role of MMP-9 in tissue destruction during TBM and its progression to the advanced stages. Specific inhibition of MMP-9 can be beneficial approach to overcome the limitations of corticosteroids with equivalent benefits for treatment of the disease.

## Abbreviations

CNS: Central nervous system
CSF: Cerebrospinal fluid
MMP-9: Matrix metalloproteinase-9
TBM: Tuberculous meningitis
TIMP-1: Tissue inhibitor of metalloproteinase-1
